# Advances in Nanodynamic Therapy for Cancer Treatment

**DOI:** 10.3390/nano14070648

**Published:** 2024-04-08

**Authors:** Bingchang Zhang, Yan Huang, Yong Huang

**Affiliations:** State Key Laboratory of Targeting Oncology, National Center for International Research of Bio-Targeting Theranostics, Guangxi Key Laboratory of Bio-Targeting Theranostics, Collaborative Innovation Center for Targeting Tumor Diagnosis and Therapy, Guangxi Medical University, Nanning 530021, China; z1573501@163.com (B.Z.); 15173164827@163.com (Y.H.)

**Keywords:** nanodynamic therapy, nanomaterials, tumor therapy, reactive oxygen species

## Abstract

Nanodynamic therapy (NDT) exerts its anti-tumor effect by activating nanosensitizers to generate large amounts of reactive oxygen species (ROS) in tumor cells. NDT enhances tumor-specific targeting and selectivity by leveraging the tumor microenvironment (TME) and mechanisms that boost anti-tumor immune responses. It also minimizes damage to surrounding healthy tissues and enhances cytotoxicity in tumor cells, showing promise in cancer treatment, with significant potential. This review covers the research progress in five major nanodynamic therapies: photodynamic therapy (PDT), electrodynamic therapy (EDT), sonodynamic therapy (SDT), radiodynamic therapy (RDT), and chemodynamic therapy (CDT), emphasizing the significant role of advanced nanotechnology in the development of NDT for anti-tumor purposes. The mechanisms, effects, and challenges faced by these NDTs are discussed, along with their respective solutions for enhancing anti-tumor efficacy, such as pH response, oxygen delivery, and combined immunotherapy. Finally, this review briefly addresses challenges in the clinical translation of NDT.

## 1. Introduction

In recent decades, there has been a steady increase in the incidence of malignant tumors, making them a leading cause of mortality worldwide. This surge in the incidence of cancer poses a significant risk to human life and well-being [1]. In oncology, notable disparities in the structural and physical characteristics of tumor tissues and their normal counterparts have attracted considerable attention [2]. These disparities are largely due to abnormal cell proliferation, metabolic activity changes, and mitochondrial dysfunction observed in tumors, among other factors. In particular, the tumor microenvironment (TME) exhibits distinct differences from the normal tissue environment and is characterized by acidic pH levels, heightened hydrogen peroxide (H_2_O_2_) production, hypoxia, and diminished catalase activity [3,4]. Such unique features not only play a crucial role in the proliferation and dissemination of tumors, but also serve as specific targets for nanodynamic therapy (NDT), aiming at the TME’s distinctive attributes [5].

With advancements in technology and in-depth medical research, various NDTs have emerged in the field of cancer treatment and are distinguished by their specificity, minimal side effects, and significant efficacy [6,7]. NDT employs nanomaterials in conjunction with specific forms of physical energy, such as acoustic [8], light [9], electric [10], radioactive [11], and chemical energies [12], to precisely target tumor cells. A hallmark of these therapies is their ability to convert local energy into mechanisms that trigger reactive oxygen species (ROS) production within the tumor, causing cell death or impairment [13]. Additionally, these therapies stimulate or bolster the body’s immune response, effectively curbing tumor growth, spread, and relapse [14].

**Figure 1:**
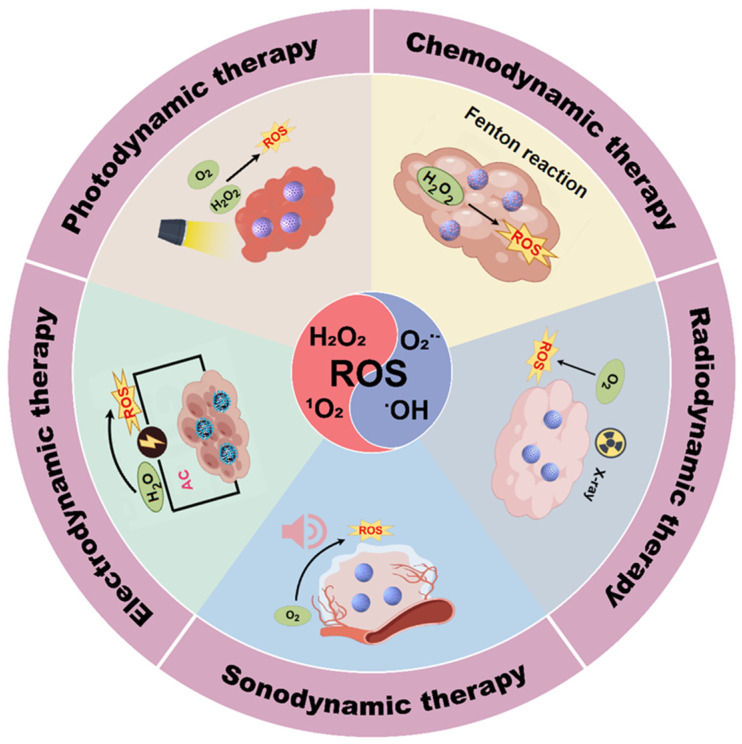
Schematic illustration of the therapeutic modalities of nanodynamic therapy (NDT), including photodynamic therapy (PDT), electrodynamic therapy (EDT), sonodynamic therapy (SDT), radiodynamic therapy (RDT), and chemodynamic therapy (CDT). By Figdraw.

Various ROS, such as H_2_O_2_ [15], singlet oxygen (^1^O_2_) [16], superoxide anions (O_2_^•−^) [17], hypochlorous acid (HClO^−^) [18], and hydroxyl radicals (•OH) [19], are crucial for cellular metabolism. ROS are naturally produced during cellular metabolic processes, and high concentrations of ROS can damage biomolecules in cancer cells, such as proteins, lipids, and DNA, thereby inducing apoptosis [20]. Several nanodynamic therapies have been developed (Figure 1), including photodynamic therapy (PDT), electrodynamic therapy (EDT), sonodynamic therapy (SDT), radiodynamic therapy (RDT), and chemotherapy (CDT) [21]. PDT utilizes the light of specific wavelengths to activate photosensitizers (PSs) within the tumor, generating ROS that directly eradicate tumor cells. Furthermore, PDT induces a local inflammatory response and modulates the immune function, which can initiate systemic anti-tumor immune reactions [22]. Through the application of an electric field, EDT disrupts tumor cells or prompts nanomaterials to produce ROS, thereby annihilating tumor cells. This electric field may also stimulate immune cells, augmenting their tumor-fighting capabilities [10]. SDT employs the mechanical and thermal effects of ultrasound to damage tumor cell structures, with the generated ROS activating the immune system for tumor cell recognition and elimination [23]. RDT utilizes X-rays emitted by radioactive substances to flood tumor cells with ROS, effectively promoting apoptosis [24]. Additionally, RDT can alter the TME to boost immune surveillance and tumor cell clearance. CDT relies on Fenton or Fenton-like reactions within the tumor zone to generate lethal substances or free radicals that directly target tumor cells for destruction [25].

Over the past few years, extensive research has been undertaken by many scholars on nanodynamic therapies aimed at combating tumors. This study spans the spectrum of innovative nanocarriers to optimize the TME and integrate various therapeutic strategies [26]. These efforts are based on catalyzing the production of reactive ROS within tumor cells and inducing tumor cell death. However, despite the significant promise of ROS-centric nanodynamic approaches in oncology, several challenges remain for their clinical application [27]. These hurdles include enhancing ROS generation, overcoming the constraints of the TME, ensuring long-term stability, and maintaining the biocompatibility of nanocarriers in vivo [28,29].

This review provides an overview of the advancements made in the field of anti-tumor mechanisms in the last five years, focusing on photodynamic, electrodynamic, sonodynamic, radiodynamic, and chemodynamic therapies. We elaborated on the design principles of nanosensitizers (Table 1) for these five types of NDTs, their anti-tumor effects, and biocompatibility. Within the realm of NDT literature, this review provides an overarching perspective of the field, highlighting the interdisciplinary and distinctive nature of current cancer research and treatment strategies. Incorporating research from the past five years, it offers a timely and comprehensive synthesis of knowledge in the rapidly advancing area of NDT, rendering it a valuable addition to the NDT literature repository. Furthermore, this review not only catalogs the latest advancements in NDT but also critically analyzes the obstacles impeding its clinical application. These challenges encompass optimizing ROS generation, overcoming the constraints imposed by the TME, ensuring the long-term stability of nanocarriers, and maintaining their biocompatibility in vivo. By juxtaposing these hurdles with potential solutions identified in prior research, this review not only sets itself apart from the existing literature but also aligns with the cutting-edge priorities in oncological research. Through this review, we aim to provide researchers with a comprehensive approach to enhance NDT and accelerate its clinical translation for tumor diagnosis and treatment. 

## 2. Photodynamic Therapy

PDT, an approved approach for cancer treatment, employs PSs and the light of specific wavelengths. It works by activating a PS with light to produce ROS that target and destroy cancer cells in the tumor region [48,49,50]. Photosensitizers, upon absorption of light, transition from a ground state to an excited singlet state. Following intersystem crossing, a more stable excited triplet state is achieved, which can interact with cellular oxygen. This interaction predominantly results in the generation of ^1^O_2_ and other ROS, initiating a cascade of oxidative damage within the cell. Subsequently, it impairs the tumor vascular network by initiating the release of vasoconstrictive agents and promoting thrombus formation [51]. Zheng et al. synthesized a highly cationic zinc phthalocyanine (Pc4) with exceptional water solubility and non-aggregating properties, which maintained high photodynamic activity in biological systems. Pc4 demonstrated profound in vitro cytotoxic effects against HepG2 cancer cells and significant in vivo anti-tumor activity. In H22 tumor-bearing mice, Pc4 demonstrated potent anti-cancer capabilities with a 98.7% tumor growth inhibition rate and strong cytotoxicity against HepG2 cancer cells in laboratory tests [52]. These results suggest that Pc4 has promising therapeutic potential for treating cancer and inhibiting tumor growth.

Moreover, PDT can trigger the release of damage-associated molecular patterns (DAMPs), leading to the immunogenic death (ICD) of tumor cells. This process activates an anti-tumor immune response that inhibits metastatic growth [53]. The production of ROS not only facilitates the direct eradication of tumor cells but also enhances the release of antigens, including calreticulin (CRT), heat shock proteins (HSPs), adenosine triphosphate (ATP), and high-mobility group box 1 (HMGB1). These antigens are then recognized by the immune system, further stimulating an immune reaction against the tumor [54,55,56,57].

**Figure 2:**
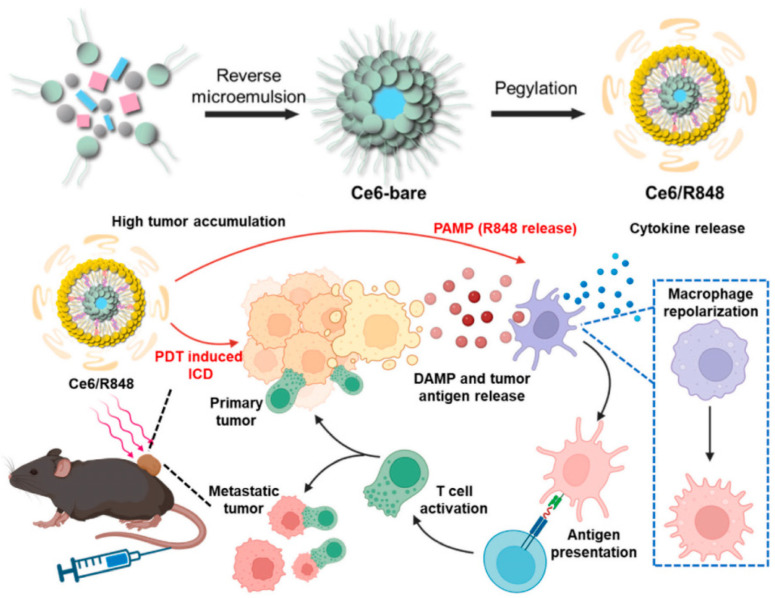
Schematic illustration of Ce6/R848 synthesis via coordination polymerization of Ce6, phosphate, and Zn^2+^ ions followed by coating with Chol-R848, cholesterol, DOPC, and DSPE-PEG2000 and schematic illustration of antitumor effects of Ce6/R848(+). Reproduced with permission from [30]. Copyright 2023, Elsevier.

Upon irradiation, PSs serve as potent inducers of ICD, specifically targeting the endoplasmic reticulum (ER) to initiate ER stress via ROS production and the subsequent release of CRT. This action facilitates the binding of extracellular ATP to the dendritic cell (DC)-specific purinergic receptors P2Y2 and P2X7, encouraging the assembly of inflammasomes and the release of pro-inflammatory cytokines [58]. Furthermore, HSPs and HMGB1 play vital roles as DAMPs in instigating ICD, where HMGB1 attaches to immature DCs and HSPs interact with the toll-like receptors TLR2 and TLR4. This interaction promotes cytokine secretion, which is crucial for the maturation of DCs and T cells, thus effectively mobilizing the immune system against tumors [59]. Jiang et al. introduced a nanoscale coordination polymer, Ce6/R848, which enhances PDT by activating toll-like receptor (TLR) 7/8. This innovative approach, through the co-delivery of the PS Ce6 and cholesterol-bound R848 (Chol-R848) to tumor sites, not only triggers ICD via Ce6-mediated PDT but also specifically targets TLR7/8 within the tumor (Figure 2). This dual action synergistically boosts the activation of antigen-presenting cells and T cells, significantly enhancing both innate and adaptive anti-tumor immune responses [30]. In experiments with the MC38 colorectal tumor mouse model, Ce6/R848 treatment achieved a 50% cure rate and suppressed tumor growth by 99.4%, significantly prolonging survival while exhibiting minimal toxicity.

Owing to the limited light penetration within tumor tissues, PDT has, to date, only been clinically utilized for the management of skin diseases and epithelial tumors [60]. To address the challenge posed by the limited penetration of light in the treatment of internal tumors, Elena et al. developed NanoLuc-miniSOG. This innovative gene encodes a structure that combines both a light source and a PS, enabling the activation of deep-tissue tumors. Demonstrated in mouse models, this “light-independent” PDT approach shows promise in curtailing tumor growth [31]. This advancement not only validates the concept of “light-independent” PDT for targeting deep-seated tumors, but also offers a novel strategy to overcome the constraints of traditional PDT methods. 

Extensive research has established that PDT can effectively initiate both innate and adaptive immune responses and that the potency of these immune reactions is linked to the oxygen levels present within tumors [61]. During PDT, the ongoing consumption of oxygen by the tumor leads to hypoxic conditions within its microenvironment, which can suppress immune activity [62]. Moreover, the tumor’s vascular abnormalities contribute to an inadequate oxygen supply, and the swift growth of tumor cells escalates the oxygen demand, markedly depleting oxygen levels in the TME compared to healthy tissues [63]. Type II PDT processes predominantly consume substantial amounts of oxygen to produce ROS that are lethal to tumor cells [64,65]. In contrast, PSs that operate through Type I reaction mechanisms require significantly less oxygen, thereby alleviating the hypoxic challenges faced by tumors during PDT [66]. To circumvent hypoxia, strategies have been devised that focus on augmenting the oxygen concentration in the TME, diminishing oxygen consumption during PDT, and developing PSs with minimal oxygen dependency to boost PDT’s therapeutic impact. 

For instance, Wang et al. developed an approach to potentiate anti-tumor effects by targeting the hypoxic TME. Their strategy merged PDT with photosynthetic microalgae (Chl), leveraging the ability of Chl under the influence of PSs to produce oxygen, thereby counteracting hypoxia. This not only improves local therapeutic outcomes and increases ROS generation, but also reverses the immunosuppressive conditions of the TME. This leads to enhanced activation and maturation of DCs, which then proceed to the lymph nodes, effectively activating CD8^+^ T cells. This activation generates a specific immune response against tumor cells and significantly enhances the anti-tumor effect in vitro [67].

**Figure 3:**
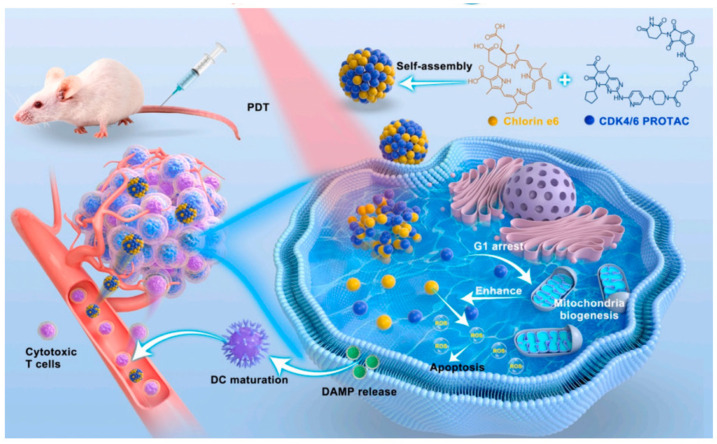
Schematic illustration of synergistic anti-tumor photodynamic therapy and immunotherapy based on CDK4/6 Nano PROTAC. Reproduced with permission [33]. Copyright 2023, Elsevier.

To amplify the anti-tumor effect, the integration of PDT with agents, such as glutathione (GSH) inhibitors, immune checkpoint blockers, chemotherapy, and radiotherapy, has been investigated. Li et al. developed an innovative nanocomposite, IRCB@M, by employing tumor cell membrane-coating techniques to encapsulate a self-assembled PS (IR-780), alongside a glutaminase inhibitor (CB-839). By hindering glutamine metabolism, this method diminishes the concentration of nicotinamide adenine dinucleotide phosphate (NADPH) and reduces GSH, consequently boosting IR-780-mediated ROS generation [32]. IRCB@M not only optimizes the TME but also augments the tumor-targeting efficiency of therapeutic agents and ensures low biotoxicity, thus increasing PDT’s effectiveness and significantly suppressing tumor proliferation both in vitro and in vivo. To enhance PDT in conjunction with chemotherapy, Sun et al. introduced a nanoparticle (NP), NP-sfb/Ce6, which simultaneously transports sorafenib and chlorin e6 (Ce6). This formulation incorporated ROS-responsive polyethylene glycolated hyperbranched polyphosphate ester for the co-encapsulation of a hydrophobic near-infrared PS (Ce6) and sorafenib. This combination facilitates the concurrent liberation of sorafenib when exposed to a 660 nm laser, intensifying the anti-tumor capabilities of PDT [68]. The strategy of the rapid release of sorafenib and low-dose PDT notably curtails tumor growth and modifies the tumor immune environment, bolstering both localized and systemic T-cell-mediated anti-tumor immunity. Moreover, tumor resistance to immunological defense can be modulated by immune checkpoints, which are composed of receptors and their corresponding ligands on neoplastic and immunological cells. Enhancing immune checkpoints within the tumor milieu can augment the ability of the immune system to detect and eradicate tumor cells. In addition, Wang et al. introduced a novel carrier-free NP that amalgamates CDK4/6 PROTAC with Ce6-mediated PDT, achieving the self-assembly of both compounds (Figure 3). This combination, with CDK4/6 PROTAC acting as a G1-S phase checkpoint inhibitor [69], reinforces mitochondria-dependent PDT and its anti-tumor immune consequences, leading to increased apoptosis and immunogenic cell death [33]. This study offers a pioneering approach by combining PDT with immune checkpoint inhibition, revealing a potent anti-tumor methodology through meticulous drug delivery and targeting of mitochondrial functions.

Despite the significant potential of PDT in cancer treatment, its application still faces challenges such as the selection of PSs, penetration depth of the light source, and durability of the treatment-induced immune response. As research on the immune mechanisms of PDT and PS light-source technology continues to advance, prospects for the application of PDT in tumor immunotherapy are becoming increasingly broad.

## 3. Electrodynamic Therapy

EDT has emerged as a cutting-edge method for oncological treatment that exploits external electric fields to precisely target and dismantle tumor cells. This therapeutic approach ingeniously integrates electrochemistry with nanotechnology, utilizing ubiquitous water molecules within the body as substrates. Under the influence of an external square-wave alternating current, ROS are continuously generated on the surface of electroactive nanoparticles [70]. Compared with traditional electrochemotherapy, EDT is not only less invasive but can also provide a uniform killing effect for larger tumors [34]. For instance, Liu et al. introduced an innovative application of platinum NPs (Pt NPs) energized by a square-wave alternating current to create a Faraday cage effect under hole doping conditions. This interaction between chloride ions and water molecules on the surface of the Pt NPs initiates the production of cytotoxic •OH, which efficiently eradicates tumor cells [10]. Nanocarriers can be used to deliver electrochemotherapy drugs and improve drug targeting and intracellular delivery efficiency. To further enhance the anti-tumor effect of EDT, Liu et al. also designed a mesoporous silica nanocomposite with Pt NPs modified on the surface and loaded it with the anti-cancer drug doxorubicin (DOX). Silica NPs modified with Pt NPs can effectively generate ROS under electric field stimulation and simultaneously release loaded DOX, thereby achieving a synergistic effect between EDT and chemotherapy [71]. Compared with single EDT or chemotherapy treatments, the use of nanocarriers loaded with chemotherapy drugs can effectively destroy tumors driven by electric fields while reducing side effects on surrounding tissues.

**Figure 4:**
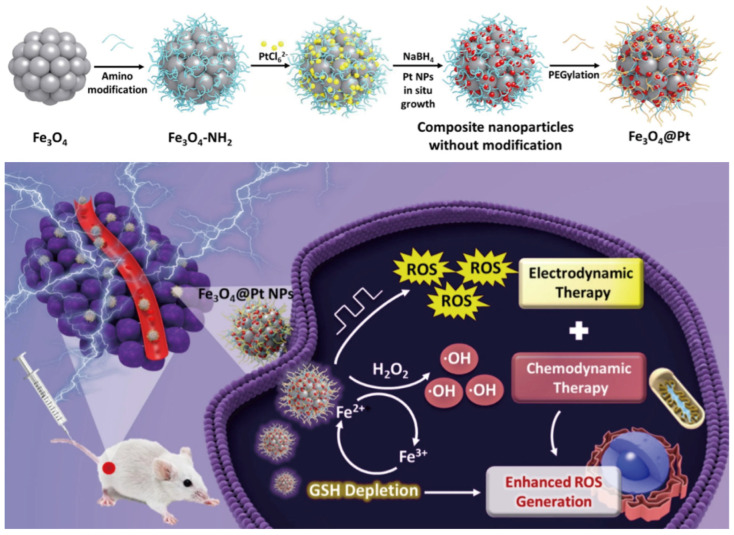
Schematic illustration of Fe_3_O_4_@Pt NPs synthesis procedure and schematic illustration of Fe_3_O_4_@Pt NPs for synergistic electrodynamic/chemodynamic tumor therapy with GSH depletion. Reproduced under the terms of the CC-BY Creative Commons Attribution 4.0 International license (https://creativecommons.org/licenses/by/4.0 (accessed on 7 March 2024)) from [73]. Copyright 2021, British Medical Council.

Despite the numerous advantages of EDT, the complexity of the TME poses significant challenges [72], particularly for ROS degradation by GSH. Chen et al. fabricated iron oxide NPs coated with Pt nanocrystals (Fe_3_O_4_@Pt NPs). When administered to the tumor location, these Pt NPs catalyzed the transformation of H_2_O_2_ into ROS under local conditions, whereas the liberated Fe^3+^ ions engaged in redox reactions to deplete GSH (Figure 4). Concurrently, under acidic tumor conditions, Fe_3_O_4_ NPs facilitate the release of Fe^2+^, which, through the Fenton reaction, catalyzes the conversion of H_2_O_2_ into more •OH. This mechanism impedes the ROS clearance capacity of tumor cells and perpetuates the generation of Fe^2+^ for enhanced •OH production [73]. By combining electrodynamics with chemodynamic therapy, this study exploited the distinctive attributes of Fe_3_O_4_@Pt NPs to boost ROS production and diminish GSH in the tumor milieu, effectively breaching the ROS defense mechanisms of the tumor. Demonstrated to possess formidable anti-tumor effectiveness in both laboratory and animal models, this strategy underscores the potential of integrated therapeutic approaches to overcome the protective barriers of the TME.

**Figure 5:**
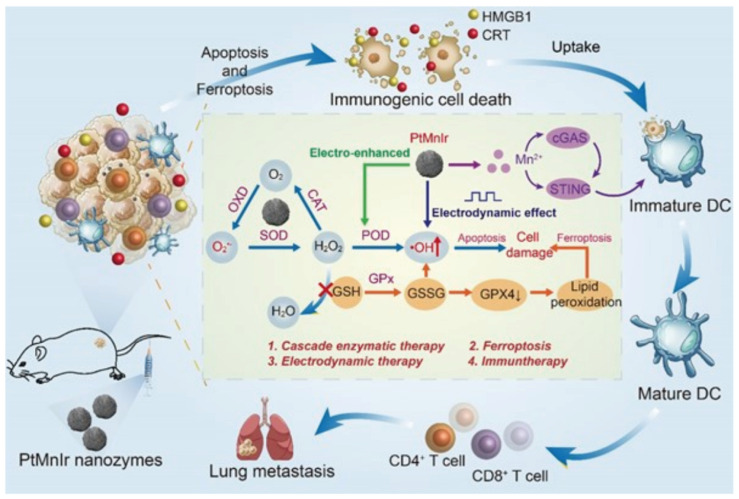
Schematic diagram of antitumor mechanism of PtMnIr nanozymes. Reproduced with permission from [36]. Copyright 2023, John Wiley and Sons.

EDT effectively induces apoptosis or necrosis in tumor cells via the application of an electric field. The immune system recognizes antigens and death-associated signals released from the deceased tumor cells, resulting in a targeted immune response. This interaction renders the integration of EDT with immunotherapy highly promising. Chen et al. designed a Pt-Pd@DON nanocarrier that effectively incorporates the glutamine antagonist 6-Diazo-5-oxo-L-norleucine (DON) into NPs for synergistic tumor treatment with EDT. EDT, through the combination of platinum–palladium NPs and electrical current, generates •OH, which directly destroys tumor cells. The concurrent administration of DON further disrupts glutamine metabolism in tumor cells, intensifying oxidative stress and leading to increased cell mortality. ICD triggered by EDT facilitates the release of DAMPs and enhances DC maturation, which in turn recruits and activates cytotoxic T lymphocytes to clear the remaining tumor cells, thereby ensuring sustained tumor growth inhibition and fostering immune memory [35]. In experiments with 4T1 tumor-bearing mice, the application of Pt-Pd@DON+E resulted in a notable reduction in tumor growth and extended the survival period. Expanding on the potential of EDT to activate immunotherapy, Li et al. have crafted a multifunctional nanocatalyst termed “Spark” PtMnIr, endowed with a spectrum of enzymatic functions such as catalase, oxidase, superoxide dismutase, peroxidase, and glutathione peroxidase. This catalyst excels at continuously generating ROS while depleting GSH through the “inner catalytic cycle.” Under the influence of EDT, the activities of Mn^3+^ and Ir^3+^ are notably enhanced, leading to GSH consumption and an increase in ROS (Figure 5). This catalytic activity results in diminished glutathione peroxidase 4 protein levels, increased lipid peroxidation in cancer cells, and the initiation of ferroptosis. Remarkably, the PtMnIr nanocatalyst also induces immune pathways and curtails distant tumor metastasis by leveraging the ICD and cGAS-STING pathways activated by Mn^2+^ ions. In the B16-F10 tumor model, this nanocatalyst demonstrated efficacious tumor suppression and immune response activation to prevent distant metastases [36]. This investigation underscores the synergistic effect of cascading enzymatic reactions, EDT, ferroptosis, and immunotherapy, revealing a potent nanomedicine strategy for combating malignant tumors.

Nanotechnology integration has significantly amplified the anti-tumor potential of EDT. An exemplary innovation involves porous platinum NPs encapsulating the chemotherapeutic agent DOX and enveloped with polyethylene glycol (PEG), forming DOX@pPt-PEG NPs. These NPs leverage electric fields to catalyze the production of ROS and employ dual mechanisms to inhibit P-glycoprotein, thereby synergistically enhancing the effects of chemotherapy alongside EDT. The ROS generated throughout the EDT process play a pivotal role in suppressing P-glycoprotein, facilitating a greater intracellular build-up of chemotherapeutic agents, and thus enhancing the overall efficacy of chemotherapy [37]. Based on comprehensive in vitro and in vivo studies, the synergistic treatment strategy of DOX@pPt-PEG NPs not only demonstrated significant anti-cancer effects in cell studies but also exhibited exceptional tumor suppression capabilities in animal models.

**Figure 6:**
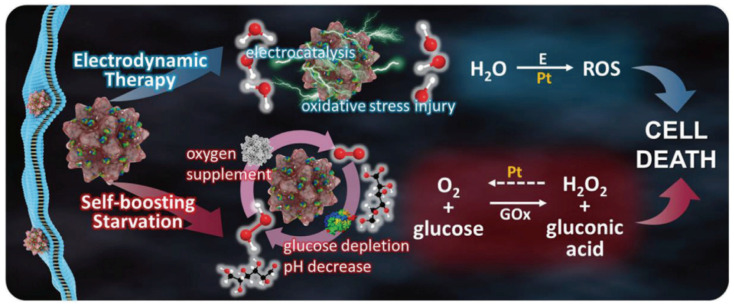
Mechanism illustration of inhibition effect induced by PtGs. Reproduced under the terms of the CC-BY Creative Commons Attribution 4.0 International license (https://creativecommons.org/licenses/by/4.0 (accessed on 7 March 2024)) from [77]. Copyright 2020, John Wiley & Sons Inc.

The rapid proliferation of tumor cells depends heavily on ample nutritional support, making the strategy of inducing nutritional deprivation by halting the glucose supply a viable method to curb tumor growth. In addition, Lu et al. prepared porous platinum nanospheres attached to glucose oxidase (GOx) enzymes (Figure 6). GOx effectively catalyzes the conversion of glucose into H_2_O_2_ [74,75], while the porous platinum nanospheres, under electric field stimulation, facilitate the decomposition of H_2_O_2_, liberating significant amounts of O_2_. This oxygen release boosts glucose consumption by GOx, leading to an induced state of tumor starvation [76]. Additionally, porous Pt nanospheres generate a substantial amount of ROS under the drive of a square-wave alternating current, inducing oxidative stress damage and prompting tumor cell apoptosis. In this study, a nanocomposite formed by combining porous Pt nanospheres with GOx achieved a 93.5% tumor cell-killing rate in vitro and significant tumor suppression in tumor-bearing mice under a square-wave alternating current [77]. This strategy, through the oxygen-induced starvation effect and the synergistic action of EDT, where the generation of ROS in EDT does not depend on the oxygen or H_2_O_2_ content in the TME, effectively achieves the ablation of tumors in a self-promoting cycle, offering an efficient new approach for tumor treatment.

EDT stands out as a forward-looking therapy for tumors, particularly those that are recurrent or resistant, and offers new avenues for patient treatment. Nonetheless, achieving this goal requires further fine-tuning of the EDT parameters and their integration with other therapeutic strategies, including chemotherapy and radiotherapy. Additionally, as nanotechnology continues to evolve and delve deeper into the mechanisms of tumor immunotherapy, the potential for incorporating EDT within this domain is expected to expand significantly. 

## 4. Sonodynamic Therapy

SDT integrates ultrasound technology with sonosensitizers to target and destroy cancer cells, while promoting immune system activation and modulating the TME [78]. As a cutting-edge tumor treatment modality, SDT primarily employs ultrasound of low frequency and intensity to activate sonosensitizers. Following ultrasonic treatment, sonosensitizers undergo a transition from the ground state to an excited state. Subsequently, this excited state of the sonosensitizer can transfer energy to the surrounding molecular oxygen, resulting in the generation of ROS that target cancer cells, with the cavitation effect identified as the principal mechanism [79,80,81]. This phenomenon encompasses the formation, growth, and bursting of microbubbles (MBs), along with persistent oscillation. In addition to cavitation, mechanisms such as thermal decomposition and sonoluminescence play a role in activating sonosensitizers for ROS production [82]. Drawing inspiration from PDT, SDT’s significant benefit is its deep tissue- and organ-penetration capabilities without the need for radiation, offering lower tissue attenuation and deep tumor-tissue penetration contingent on the ultrasound frequency used. To explore the therapeutic potential of SDT, researchers have developed an innovative compound, platinum(II)-indocyanine (Pt-Cy), capable of generating ^1^O_2_ upon ultrasound or light exposure, which shows effective US-induced cytotoxicity against breast cancer 4T1 cells. Metabolomic studies further confirmed that Pt-Cy diminishes the cellular levels of GSH and glutathione peroxidase 4, leading to the induction of ferroptosis in 4T1 cells [38]. In vivo studies have shown that Pt-Cy effectively inhibits tumor growth when combined with ultrasound irradiation. The synergistic effect of SDT using Pt-Cy was found to be even more effective in reducing tumor size than using PDT alone.

In the quiescent state, sonosensitizers are benign. However, they transform into cytotoxic agents when activated by ultrasound, generating ROS through mechanisms such as sonocavitation, sonoluminescence, or pyrolysis [83]. Investigations into sonosensitizers have delved into various compounds such as porphyrins, phthalocyanines, and selected organic dyes. Studies have aimed to identify sonosensitizers that combine efficiency, minimal toxicity, and precise targeting to bolster the safety and effectiveness of SDT in cancer treatment [39]. In recent years, researchers have incorporated sonosensitizers into nanomaterials to significantly improve the targeting and cytotoxic effects of SDT on tumors. For example, Fan et al. constructed a novel sonosensitizer based on carbon dots (C-dots) and lipid-shell gas MBs, called C-dot MBs. In vitro experiments indicated that ROS were effectively produced by C-dot MBs when exposed to ultrasound at a frequency of 1 MHz. This results in peroxidation of the cell membrane, elevated levels of intracellular ROS, and, ultimately, cell apoptosis. When utilized in the TRAMP tumor-bearing mouse model, the combined treatment with C-dot MBs and ultrasound increased the rate of ROS-induced cell damage and apoptosis by factors of 3 and 2.5, respectively, compared to treatment with C-dot MBs alone [40]. Additionally, Chen et al. developed a novel sonodynamic-theranostic platform by merging gold NPs (Au NPs), manganese dioxide (MnO_2_), and biodegradable ultrathin two-dimensional black phosphorus (BP) nanosheets to create a Au/BP@MS composite. The attachment of Au NPs to BP nanosheets enhanced the electron-hole pair separation efficiency by reducing BP’s bandgap, thus amplifying its sonodynamic action. The MnO_2_ component catalyzes the decomposition of endogenous H_2_O_2_ in the TME, boosting oxygen levels and depleting GSH. This action heightens oxidative stress in tumors, significantly increases ROS production, and inhibits tumor growth [84]. In essence, this study explored the immense potential of Au/BP as a sonosensitizer, offering a fresh perspective on the development of tumor therapy-diagnostic nanoplatforms based on BP nanosheets.

Moreover, SDT is known to trigger ICD, releasing DAMPs and pro-inflammatory cytokines, which, in turn, activate mature DCs, ultimately eliciting T cell-driven immune responses that target tumors for destruction [85]. In a novel approach, Li et al. engineered 2,2′-azobis [2-(2-imidazolin-2-yl)propane] dihydrochloride (AIPH) onto platinum–zirconium oxide nanosonosensitizers, creating the ZrO_2−x_@Pt/AIPH (ZPA) nanocomposite. Upon ultrasound irradiation, ZPA enhances the production of ^1^O_2_ and •OH in tumor cells, whereas AIPH’s thermal decomposition under ultrasound irradiation generates cytotoxic alkyl radicals (•R). This combination of ROS and thermally induced alkyl radicals leads to irreversible degradation of tumor cells. In cellular experiments, the “ZPA + US + H_2_O_2_” treatment resulted in the strongest green fluorescence of CRT on the membrane of 4T1 cells, the highest level of HMGB1 fluorescence attenuation, and the lowest intracellular ATP levels, with the highest extracellular ATP content [41]. These results suggest that under the enhanced action of SDT, ZPA induces ICD, triggering the secretion of multiple DAMPs, which is conducive to reversing immune suppression and improving anti-tumor efficacy.

**Figure 7:**
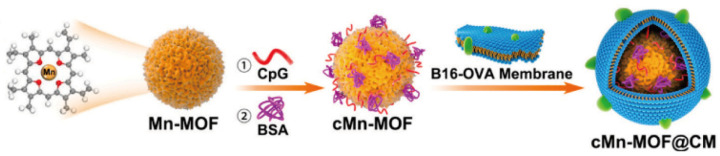
Schematic illustration of the preparation of cMn-MOF@CM. Reproduced with permission from [88]. Copyright 2021, Elsevier.

Nanocarriers can be designed to simultaneously carry sonosensitizers and immunostimulatory molecules, such as CpG oligodeoxynucleotides. This design aims to kill tumor cells through SDT while activating toll-like receptor 9 with CpG, further activating DCs and T cells to enhance the anti-tumor immune response [86]. Lin et al. developed a multifaceted nanosonosensitizer, TiO_2_-Ce6-CpG, by amalgamating titanium dioxide (TiO_2_) NPs with the sonosensitizer Ce6 and immune adjuvant CpG oligodeoxynucleotides. This complex not only bolsters the effectiveness of SDT in curtailing primary tumor growth, but also amplifies the immune response via CpG, eliciting a robust anti-tumor immune reaction [87]. In addition, metal–organic frameworks (MOFs) have been crafted as versatile platforms for carrying immune modulators, including immune checkpoint inhibitors or molecules that stimulate immunity, creating a combined force with SDT. Zhan et al. synthesized a composite material, cMn-MOF@CM (Figure 7), by integrating a manganese-based MOF (Mn-MOF) with the immune adjuvant CpG and encasing it in the cell membrane from melanoma B16 cells expressing ovalbumin. This composition has been shown to mitigate hypoxic conditions within tumors through its sonodynamic activity and to foster immunogenic cell death, thereby enhancing the immune reaction against tumor cells [88]. When applied in conjunction with anti-PD-1 agents, it potentially has a synergistic effect on tumor suppression and the establishment of a lasting immune memory, thus inhibiting tumor progression and recurrence.

**Figure 8:**
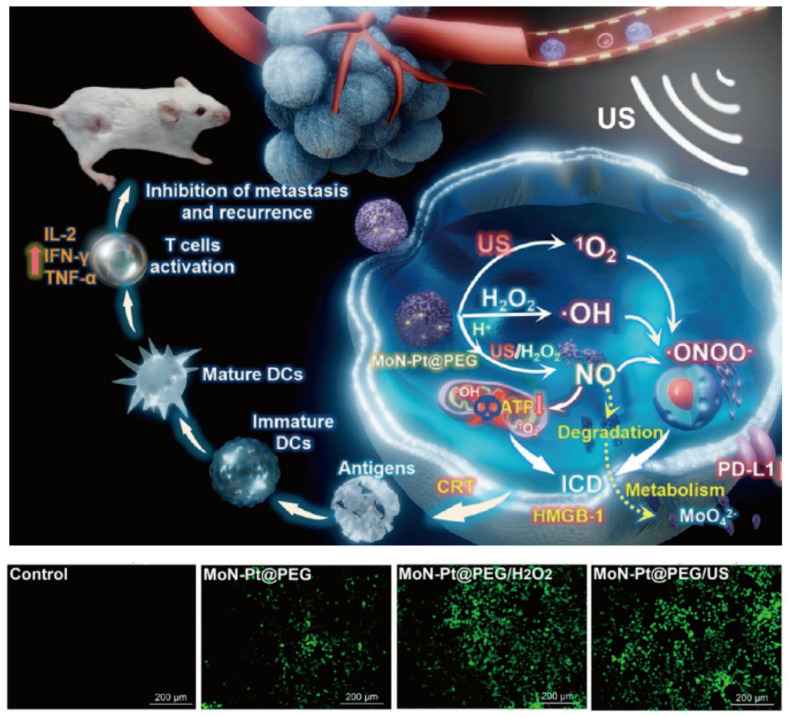
Schematic illustration of synergetic NO therapy and SDT from MoN-Pt@PEG for anticancer and immune activation and fluorescence images of DCFH-DA staining 4T1 cells incubated with MoN-Pt@PEG under different conditions. Reproduced with permission from [91]. Copyright 2023, American Chemical Society.

Furthermore, endogenous gasotransmitters, such as nitric oxide (NO) and carbon monoxide (CO), act in concert with SDT to fight tumors effectively when elevated [89]. This synergy occurs through various mechanisms, including mitochondrial and DNA damage, DNA repair inhibition, cellular respiration suppression, and inflammation amplification [90]. Gas-assisted therapy, when combined with SDT, has been proposed to augment the therapeutic impact. Bai et al. employed porous molybdenum nitride (MoN) nanospheres as effective NO donors enveloped in PEG to create MoN-Pt@PEG NPs. This nanocomplex, under ultrasound, selectively releases NO in the tumor milieu, fostering the in situ formation of cytotoxic peroxynitrite (•ONOO^−^), while also serving as a sonosensitizer in SDT by initiating ROS production, which damages tumor cells (Figure 8) [91]. As demonstrated in both in vitro and in vivo studies, this nanosystem showed superior anti-cancer efficacy and induced immune activity, directly addressing tumor metastasis and recurrence. In addition, this nanosystem exhibited excellent biocompatibility and metabolic characteristics in vivo.

SDT, when integrated with nanomaterials, shows significant promise in tumor immunotherapy by inducing ICD and activating the immune system, facilitating therapeutic outcomes not only in the treated area, but also systemically. Nonetheless, for SDT to transition into clinical practice, several hurdles must be overcome. These include refining sonosensitizers for better selectivity and therapeutic outcomes, standardizing ultrasound parameters for uniform treatment results, and conducting extensive clinical trials to validate their safety and effectiveness in various cancer types.

## 5. Radiodynamic Therapy

RDT emerges as a groundbreaking approach in cancer treatment, skillfully merging radiation therapy’s principles with those of PDT [92]. Upon exposure to X-ray irradiation, PS transitions from its ground state to an excited state, during which electrons are transferred to the surrounding substratum, resulting in the production of radical ions. Subsequently, these radical ions can react with oxygen to generate ROS, facilitating apoptosis [93]. RDT’s key advantage over traditional PDT lies in its superior tissue-penetration capabilities, enabling the effective treatment of tumors located deep within the tissues [94]. Research on RDT’s anti-tumor actions has revealed its dual capacity to induce tumor cell death and disrupt tumor microvasculature, while provoking a pronounced local inflammatory response [95]. ROS generated from the interaction between PS and X-rays target tumor cells, modify their biological behaviors toward apoptosis, and concurrently invigorate the immune system. This activation allows immune cells to recognize and eliminate tumor cells, thereby delivering a comprehensive approach for cancer treatment [42,96,97].

**Figure 9:**
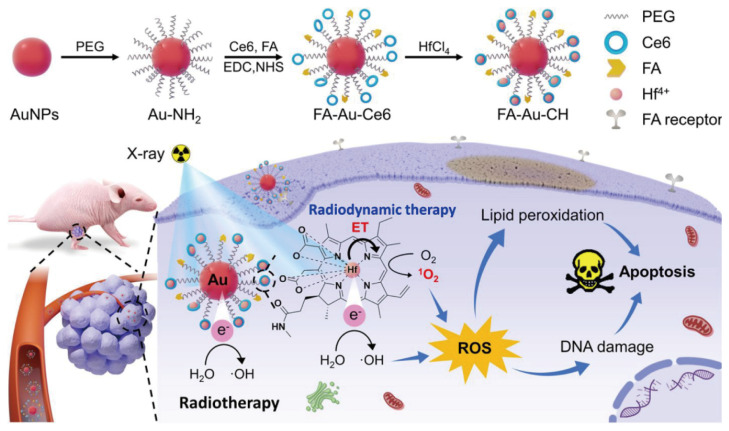
Illustration of fabrication of FA-Au-CH nanosensitizer and the detailed working mechanism of RT-RDT for tumor treatment. Reproduced with permission from [99]. Copyright 2023, American Chemical Society.

As a typical example, an intelligent nanotherapeutic system composed of hafnium (Hf)-chelated porphyrin-modified gold radiosensitizers, PS Ce6, and folic acid (FA), FA-Au-CH, combined with RDT, enhances the treatment of colon cancer (Figure 9). Owing to the strong X-ray absorption properties of Au and Hf, as well as their capacity to transmit radiation energy to Ce6, resulting in the generation of ROS [98], FA-Au-CH NPs are capable of selectively accumulating in cancer cells via endocytosis mediated by folate receptors. This process enhances RDT by simultaneously triggering radiosensitization and radiodynamics. This is based on the ability of Hf^4+^ to transfer radiative energy to the PS, effectively generating ROS under a single X-ray irradiation. In experiments assessing the generation of ROS in HCT116 cells, strong green fluorescence was observed when the cells were exposed to FA-Au-CH NPs and X-rays, indicating the significant presence of ROS. Additionally, cells treated with FA-Au-CH and X-ray irradiation exhibited lipid peroxidation levels 2.42 times higher than those treated with FA-Au-Hf alone [99]. These results highlight the ability of FA-Au-CH NPs to target and induce ROS production in cancer cells, culminating in their death. In vivo studies further confirmed the tumor-targeting efficacy of the system, with computed tomography (CT)-imaging signal intensities at tumor sites in mice peaking at 24 h and showing a 1.24-fold increase over the control Au-CH group. These findings provide strong evidence of the effective tumor-targeting capability of FA-Au-CH, highlighting its potential for in vivo tumor CT imaging.

The effective deployment of RDT faces significant challenges owing to the hypoxic conditions within the TME, which result in the reduced production of ROS, thereby diminishing RDT’s capability to combat tumors. To address the issue of treating deeply located and hypoxic tumors, Clement et al. developed PLGA-VP-PFOB NPs by incorporating verteporfin (VP) and perfluorooctyl bromide into biodegradable NPs made from poly(lactic-co-glycolic acid) (PLGA). These NPs were specifically designed to bolster the efficacy of RDT against cancer by boosting ROS generation in situ, even when exposed to low-dose radiation and in the absence of oxygen [100]. In vitro experiments under simulated hypoxic conditions demonstrated that these NPs exhibited high cytotoxicity against the human pancreatic cancer cell line PANC-1 upon X-ray stimulation. Additionally, dihydrolipoic acid-coated gold nanoclusters (AuNC@DHLA) have been used in RDT for direct X-ray absorption without the need for secondary scintillators or PSs. The operation of AuNC@DHLA relies on electron transfer mechanisms that catalyze the formation of specific ROS types, namely O_2_^•−^ and •OH. This novel strategy facilitates the production of a surplus of ROS, even in oxygen-deficient environments. This overcomes challenges associated with suboptimal energy transference and hypoxic conditions prevalent within the TME, which impede conventional RDT techniques that utilize scintillator-based approaches [43]. Similarly, to overcome hypoxia in the TME, Fan et al. developed hollow mesoporous organosilica NPs engineered for the effective carriage and delivery of tert-butyl hydroperoxide (TBHP) and pentacarbonyliron (Fe(CO)_5_). Upon X-ray exposure, these NPs triggered the activation of peroxide bonds within TBHP, generating •OH radicals in an oxygen-independent manner, while concurrently liberating CO molecules from Fe(CO)_5_ [101]. Research conducted in vitro and in vivo has demonstrated that •OH and CO produced by this method significantly inhibit tumor growth under both oxygen-rich and oxygen-poor conditions.

**Figure 10:**
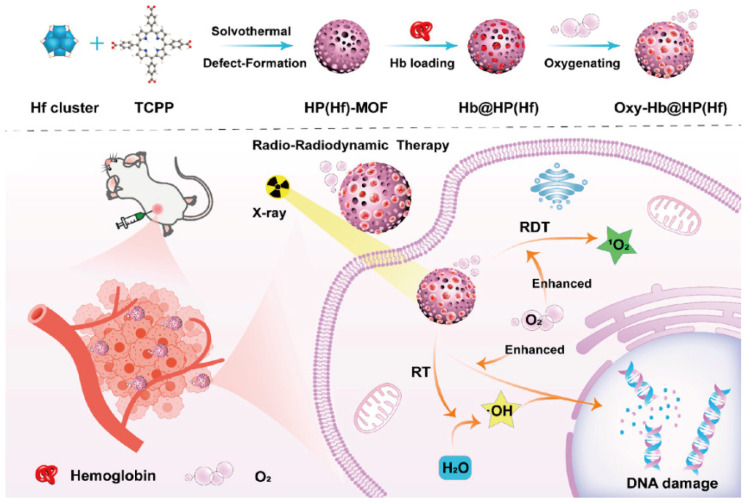
Reproduced with permission from [44]. Copyright 2023, American Chemical Society.

In addition to developing NPs that home in on tumors under hypoxic conditions, an alternative approach to alleviate tumor hypoxia involves the direct delivery of oxygen to the tumor site through the use of nanomaterials. The direct application of hemoglobin (Hb), the primary oxygen-carrying protein in red blood cells, is restricted because of potential nephrotoxicity and immunogenicity issues. To circumvent these challenges, researchers have developed multifaceted nanosystems that not only encapsulate Hb for oxygen delivery but also enhance radiation therapy as radiosensitizers. These innovative systems preserve the oxygen transport capability of Hb while minimizing its adverse effects, thus effectively managing tumor hypoxia [102]. For example, Zhao et al. engineered a nanosensitizer utilizing a MOF, dubbed Hb@HP(Hf) (Figure 10). This pioneering nanosensitizer incorporated Hf, which captures X-rays and emits energy in the form of visible light. This energy emission activates the photosensitive tetrakis(4-carboxyphenyl) porphyrin, facilitating the production of ROS. Additionally, Hb@HP(Hf) can deliver a substantial oxygen payload, significantly mitigating the oxygen-deficient conditions of the TME and boosting the efficacy of RDT [44]. Further in vivo studies showed that the HP(Hf) MOF improved the hypoxic environment within solid tumors, resulting in excellent anti-tumor effects.

After mitigating hypoxia within the TME, ROS generated by RDT trigger an extensive immune response against tumors. This effect was significantly amplified when RDT was combined with immune checkpoint inhibitors, facilitating the complete elimination of both primary and metastatic tumors. Utilizing NPs such as Hb@Hf-Ce6 has been shown to enhance RDT’s effectiveness, improve the management of the hypoxic TME, and stimulate immune responses against tumors in synergy with PD-1 immune checkpoint inhibitors [103]. Hf within these NPs serves as a radiosensitizer, increasing radiation absorption, and thereby elevating ROS production, which contributes to the suppression of primary tumor growth. Concurrently, blockade of the PD-1 pathway addresses the challenge of insufficient immune responses, often observed with RDT alone [104]. In vivo experiments showed that combined treatment with Hb@Hf-Ce6NPs+PD-1(+) inhibited hypoxia-induced immunosuppressive M2 phenotype polarization effectively eliminated regulatory T cell activity, and induced the maturation of effector T cells (CD3^+^ and CD8^+^) in both distant and primary tumors. In a mouse model of lung metastasis, the NP+PD-1(+) group showed delayed cancer cell metastasis without death, indicating that RDT combined with Hb@Hf-Ce6 NPs immunotherapy effectively inhibited lung tumor cell metastasis. Hb@Hf-Ce6 NPs, by reactivating immune cells and releasing cytokines, maintain and promote the proliferation of immune cells, thereby enhancing RDT immunotherapy for both primary and distant tumors.

In summary, RDT offers a new strategy for improving cancer treatment outcomes. In particular, when combined with immune checkpoint blockade, this strategy can activate the immune system to combat tumor growth and metastasis. Future research should continue exploring the application of different PSs, optimizing RDT treatment parameters, and evaluating synergistic effects with other treatment methods to achieve more effective and safer tumor-treatment protocols.

## 6. Chemodynamic Therapy

CDT is an advanced cancer treatment that induces cell apoptosis by generating hydroxyl radicals within the tumor region through Fenton or Fenton-like reactions, ultimately suppressing tumor growth [105]. The core of the Fenton reaction is the chain reaction between iron ions (Fe^2+^) and H_2_O_2_, which promotes the production of hydroxyl radicals. Recent studies have revealed that metal ions such as Cu^+^, Mn^2+^, Co^2+^, and Ti^3+^ can also effectively catalyze H_2_O_2_ to produce •OH through mechanisms akin to Fenton reactions [45,46,106]. Owing to varying catalytic conditions, different chemodynamic catalysts exhibit distinct effects in cancer therapy. Amidst the rapid advancement of Fenton and Fenton-like nanomaterials, CDT has attracted considerable interest owing to its distinctive benefits, including elevated tumor selectivity, minimal adverse effects [107], therapeutic procedures that obviate the need for external-field activation, and the capability of modulating hypoxic conditions and the TME [108].

However, the success of CDT is constrained by several factors, including the challenge of delivering therapeutic agents efficiently and the complexity of the TME. Firstly, a low concentration of H_2_O_2_ within tumor cells can diminish the effectiveness of CDT [109]; secondly, there is a risk of metal ion depletion en route to cancer cells, and potential harm to healthy cells. Thirdly, to date, only a limited selection of metal elements are recognized as both effective and safe for CDT, leaving the potential of numerous other metals untapped. Finally, the effectiveness of many CDT agents is often limited to acidic conditions, presenting a challenge for their broader application in cancer therapy [110,111].

Outside the TME, owing to mildly alkaline conditions and insufficient hydrogen, the capability of H_2_O_2_ of catalyzing •OH is significantly lower, ensuring the safety of the surrounding normal tissues [112]. Nonetheless, the scarcity of endogenous H_2_O_2_ poses a significant hurdle in achieving total tumor eradication, even when employing Fenton reactions. To address the challenge posed by inadequate endogenous H_2_O_2_, Yang et al. introduced a synergistic strategy that merged CDT with starvation therapy to suppress tumor growth. They synthesized a multifunctional nanocomposite, SC@G, by integrating GOx with strontium copper silicate (SrCuSi_4_O_10_). This composite exploited GOx to convert the glucose present within the tumors into gluconic acid, thereby generating H_2_O_2_ in situ. This acidification of the TME accelerated the breakdown of SrCuSi_4_O_10_, resulting in the release of Sr and Cu ions. These ions then catalyzed the conversion of H_2_O_2_ into the more harmful •OH, forging a dual-attack mechanism against cancer through starvation therapy and CDT [113]. Moreover, the photothermal effect generated under laser irradiation further enhanced GOx activity, accelerating the intratumoral CDT reactivity. SC@G not only initiates starvation therapy, but also produces H_2_O_2_ in quantities sufficient for effective CDT. Laboratory tests using laser irradiation at 808 nm and 1064 nm demonstrated that the viability of 4T1 cancer cells decreased by 18.7% and 16.4%, respectively. In experiments involving a 4T1 tumor-bearing mouse model, the group treated with SC@G and a 1064 nm laser achieved total tumor elimination, showing that the combined impact of photothermal therapy, starvation therapy, and CDT surpasses the efficacy of any single treatment method. Cisplatin has been shown to specifically trigger the activation of NADPH oxidase in cancer cells. This enzyme catalyzes the transfer of electrons from NADPH to O_2_, producing the superoxide anion (O_2_^•−^), which superoxide dismutase subsequently converts into H_2_O_2._ Building on this, Ren et al. formulated PTCG NPs incorporating epigallocatechin gallate, a phenolate platinum(IV) prodrug, and a polyphenol-modified block copolymer to achieve high drug-load efficiency [114]. Once released inside the cell, activated cisplatin elevates intracellular H_2_O_2_ levels, setting off a series of reactions that leverage the Fenton reaction to generate highly toxic ROS, thus facilitating potent CDT.

**Figure 11:**
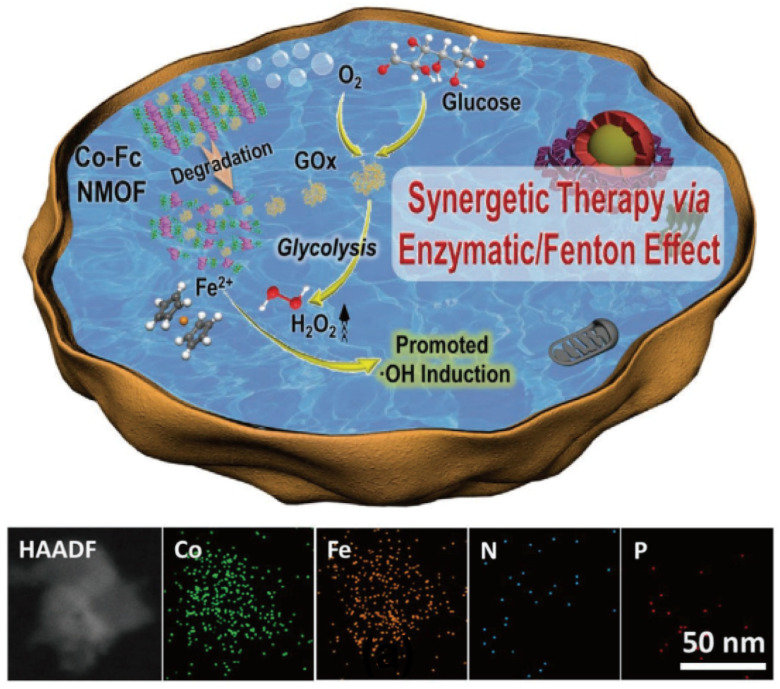
Schematic illustration of Co-Fc@GOx as a cascade enzymatic/Fenton reaction platform for promoted •OH induction and EDS element mapping of Co-Fc@GOx. Reproduced with permission from [117]. Copyright 2020, John Wiley and Sons.

Owing to their unique properties, MOFs have shown tremendous application potential in the field of CDT [115]. In particular, the stimuli-responsive and porous structure of MOFs make them ideal carriers for effective drug delivery, for targeting the TME [116]. Fang et al. merged a cobalt–ferrocene MOF (Co-Fc NMOF) with GOx to create a Co-Fc@GOx nanocomposite. This composite serves not only as an adept carrier, facilitating the targeted delivery of GOx into the TME, but also engages in the generation of highly toxic •OH, via the Fenton reaction (Figure 11). Within the TME, the GOx component of the Co-Fc NMOF accelerates the conversion of glucose into gluconic acid and H_2_O_2_, thereby increasing the intracellular acidity and H_2_O_2_ concentration. This establishes a setting for the Fenton reaction of the Co-Fc NMOF, which ultimately enhances ROS production [117]. Unlike Co-Fc NMOF alone, the Co-Fc@GOx variant incorporates P from the GOx molecules, adding a novel element to its composition. Through various in vitro assessments, including CCK-8 assays, live/dead cell staining, and flow cytometry, it was established that Co-Fc@GOx markedly suppressed the viability of 4T1 cancer cells more effectively than Co-Fc NMOF alone, with the cell-destructive impact being amplified with increasing GOx concentrations. In vivo studies in a 4T1 mouse tumor model revealed that Co-Fc@GOx not only significantly curtailed tumor growth but also maintained exceptional biocompatibility and biosafety, showing no detrimental effects on healthy tissues.

**Figure 12:**
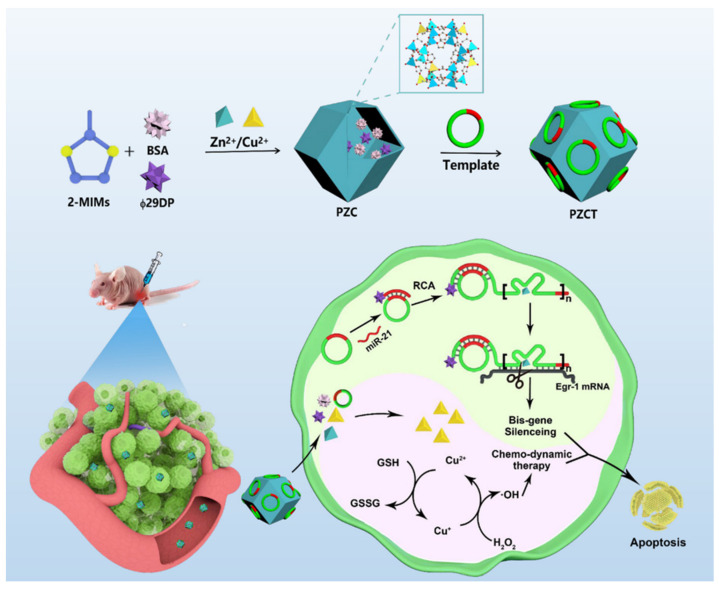
The synthesis of PZCT nanoparticles and illustration of PZCT nanosystem for intracellular RCA-assisted DNAzyme-based gene therapy combined with chemo-dynamic therapy. Reproduced with permission from [47]. Copyright 2024, American Chemical Society.

The Fenton reaction involving Fe^2+^ relies on a low-pH environment, which hinders the generation of ROS within tumors, to some extent. In a mildly acidic TME, metal ions such as Cu^+^ and Mn^2+^ have been shown to catalyze the conversion of H_2_O_2_ into •OH more effectively than Fe^2+^ [118]. Therefore, Huang et al. designed a Zn^2+^/Cu^2+^ bimetallic MOF loaded with a rolling-circle-amplification substrate, PZCT, for precise and efficient combined gene therapy using DNAzymes and CDT. The activation process of PZCT is controlled by a tumor-specific bioreciprocal mechanism overexpressing miR-21, which utilizes the catalytic action of Zn^2+^ to generate a large amount of DNAzymes, silencing EGR-1 mRNA, and thereby inhibiting tumor growth (Figure 12). Simultaneously, Cu^2+^ doped within PZCT reduces GSH and converts endogenous H_2_O_2_ into toxic •OH, thereby exerting CDT effects [47]. This dual strategy of gene therapy combined with CDT demonstrated significant tumor-eradication effects in vivo. Another study demonstrated a pH-independent Fenton-like reaction strategy to facilitate the generation of •OH within tumors. Chen et al. constructed ultrasmall bovine serum albumin (BSA)-modified chalcopyrite NPs (BSA-CuFeS_2_ NPs). According to the 3,3′,5,5′-tetramethylbenzidine colorimetric assay, BSA-CuFeS2 produced •OH concentrations without significant differences under various pH conditions, proving its ability to perform pH-independent Fenton-like reactions, efficiently generating •OH within the mildly acidic TME. BSA-CuFeS2 NPs also demonstrated a high photothermal conversion efficiency (38.8%), indicating promising prospects for combining photothermal therapy with CDT to synergistically kill tumor cells and inhibit tumor growth [119].

In summary, CDT, as a non-invasive anti-tumor treatment modality, has shown broad application prospects owing to its unique mechanism of action and therapeutic potential. Through continuous research and optimization of nanocarriers to overcome the limitations of the TME in CDT, as well as exploring effective combinations of CDT with other treatment modalities (such as immunotherapy and targeted therapy), CDT is evolving into a more effective and safer cancer-treatment option, offering renewed hope for patients with cancer.

## 7. Role of Nanomedicine in NDT

Amidst the ongoing development of nanotechnology within the medical sector, nanomedicines (Table 2) constructed by researchers leveraging the unique physical, chemical, and biological properties of nanomaterials have garnered widespread attention and research interest in tumor therapy [120]. By nanonizing drugs, nanomedicines can significantly enhance the pharmacokinetic properties of therapeutic agents, thereby augmenting their efficacy. This is achieved through precise delivery, sustained and controlled release, multimodal therapy, and excellent biocompatibility. Such advancements facilitate efficient drug delivery to tumors, optimize drug distribution within organisms, and improve therapeutic outcomes, while minimizing adverse effects on normal tissues [121,122]. However, the metabolism and excretion mechanisms of nanomedicines differ markedly from those of traditional drugs, potentially leading to accumulation within organisms and consequent long-term toxicity issues [123]. Furthermore, the size and surface characteristics of nanoparticles may influence their ability to navigate through hepatic, renal, or other excretory pathways, affecting not only the drug clearance rate but also the overall safety and efficacy of the therapeutic regimen [124].

In the domain of tumor therapy, nanomedicines predominantly operate through two major mechanisms: thermodynamics [125] and kinetics [126]. A commonality among NDT modalities is their capability of transforming local energy into a mechanism that activates the production of ROS within the tumor, thereby inducing cellular death or damage. Thus, ROS play an indispensable role in the kinetic approach to tumor treatment [127]. Regrettably, NDT primarily relies on oxygen or endogenous H_2_O_2_ to generate •OH, not only limiting the efficiency of ROS production but also potentially exacerbating the hypoxic condition within tumor regions, which fundamentally contributes to tumor resistance to NDT [128]. Given that NDT chiefly utilizes nanocarriers as drug delivery systems, it achieves targeted drug delivery, controlled release, and responsive therapy for the tumor microenvironment by regulating the kinetic behavior of drugs in vivo [129]. To expedite the development and clinical translation of NDT, research should be directed towards the following: (1) developing multifunctional nanoplatforms capable of simultaneous drug delivery, NDT, and imaging, to facilitate integrated disease diagnosis and treatment; (2) designing intelligent nanocarriers that respond to changes in the TME, such as pH, temperature, or enzyme activity, for precise control over the release and activation of nanomedicines; and (3) addressing tumor hypoxia in NDT through the use of nanocarriers capable of carrying or generating oxygen to enhance the efficacy of NDT in treating deep-seated tumors.

## 8. Conclusions and Outlook

This article provides an overview of new technologies for nanodynamic anti-tumor therapy and discusses the potential of five NDTs in cancer treatment: photodynamic, electrodynamic, sonodynamic, radiodynamic, and chemodynamic therapies. However, because each therapy has its own shortcomings, they cannot achieve the desired effect when used alone. Consequently, contemporary research is increasingly directed toward integrating multiple NDT modalities and incorporating immunotherapy to devise comprehensive multimodal tumor treatment stratesgies that enhance therapeutic outcomes. Despite significant progress in experimental research, only PDT has been approved as a first-line treatment for non-melanoma skin cancer. However, clinical translation based on NDT faces significant challenges.

A critical barrier to the clinical adoption of NDTs is the absence of clinically validated medical devices for their application, and most NDT-triggering mechanisms are currently classified as experimental tools. This limitation confines NDT research to preclinical and animal-based studies and impedes the progression of human trials. For NDTs to transition from research laboratories to clinical practice, the development and standardization of safe and effective medical devices tailored for NDT applications is imperative.

Additionally, NDT relies on nanomaterials with specific properties that target tumor cells and induce therapeutic effects. However, the biocompatibility and biodegradability of NDT nanomaterials such as nanocarriers containing PSs, sonosensitizers, and MOFs require further investigation. The biological effects and biosafety data of nanomaterials derived from animal experiments are insufficient to ensure the safe transformation and use of nanomaterials from experimental animals to humans. The development of safe and efficient nanomaterials is crucial for realizing the clinical application of NDT technology, which requires scientific researchers to not only pay attention to the therapeutic efficacy of nanomaterials, but also to conduct in-depth research on their safety in the human body. Moreover, the therapeutic mechanisms of NDT are not well understood, posing challenges in achieving multimodal treatment. Although laboratory studies have shown the potential of multimodal NDT technology in combating tumors, further research is needed to effectively integrate these technologies while ensuring patient safety, thereby enhancing treatment efficacy. Addressing these key aspects is crucial for the clinical translation of NDT.

Although NDT technologies still face multifaceted challenges in clinical applications, they have shown tremendous potential in cancer treatment. Future research aimed at ensuring biosafety must continually optimize the design of nanocarriers with advancements in technology and deepen interdisciplinary collaboration. Moreover, the development of multimodal treatment modalities in conjunction with immunotherapy is crucial for achieving comprehensive tumor eradication. We believe that NDT can be applied to clinical oncology treatments in the future.

## Figures and Tables

**Table 1:** Summary table of the therapeutic properties of various Nanosensitizers for NDT.

Nanosensitizers	Nanodynamic Therapy (NDT)	Therapeutic Properties	Ref.
Ce6/R848	PDT	Ce6-mediated PDT releases tumor antigens, delivers R848-activated TLR7/8 into tumors, and enhances anti-tumor immune responses.	[30]
NanoLuc-miniSOG	PDT	Gene encoding activates PDT to generate ROS for treating deep-seated tumors.	[31]
IRCB@M	PDT	Reducing oxygen consumption, blocking GSH, preventing the depletion of ROS, and enhancing the therapeutic efficacy of PDT.	[32]
PROTAC/Ce6	PDT	The ROS generated by PDT induce ICD, triggering an anti-tumor immune response.	[33]
KCCP	EDT	In the presence of chlorine, the electric field activates Pt NPs and Ca^2+^ to produce ROS, enhancing tumor suppression.	[34]
Pt-Pd@DON	EDT	EDT combined with immunotherapy synergistically suppresses tumor growth.	[35]
PtMnIr	EDT	Nanocatalysts in combination with EDT continuously generate ROS while depleting GSH, promoting ferroptosis and apoptosis in tumor cells.	[36]
DOX@pPt-PEG	EDT	ROS produced by the combination of EDT and chemotherapy can induce the inhibition of P-gp, thereby synergistically suppressing tumor growth.	[37]
Pt-Cy	SDT	Under US irradiation, ^1^O_2_ is generated, inducing ferroptosis and inhibiting tumor growth.	[38]
Cu(II)NS	SDT	Overexpressed GSH in the TME reduces Cu^2+^ to Cu^+^, enabling the production of ROS in SDT.	[39]
C-dots MBs	SDT	Following US irradiation, the generation of ROS enhances tumor therapy.	[40]
ZrO_2−x_@Pt	SDT	With the aid of immune checkpoint blockade, sonodynamic-thermotherapy is combined for the treatment of tumors.	[41]
Hf-DBP-Pt	RDT	ROS generation via RT-RDT remodels the TME, facilitating neutrophil-mediated tumor regression.	[42]
AuNC@DHLA	RDT	Without the need for additional scintillators or photosensitizers, excessive ROS production under hypoxic conditions leads to significant therapeutic effects on solid tumors in vivo.	[43]
Hb@HP(Hf)	RDT	Massive oxygen delivery effectively alleviates hypoxia in the TME, thereby greatly enhancing the tumor treatment efficiency of RT-RDT.	[44]
MCBR	CDT	The Fenton-like reaction generates •OH, synergizing photothermal therapy, CDT, and immunotherapy for anti-tumor activity.	[45]
PLGA-SPIO& Vc	CDT	CDT combined with SDT, where Vc decomposes into H_2_O_2_, creates favorable conditions for the Fenton-like reaction, enabling continuous generation of ROS, thereby enhancing the anti-tumor effect.	[46]
NMOF ZIF-8	CDT	The precise and efficient combination of gene and CDT significantly demonstrates tumor eradication effects.	[47]

**Table 2:** Summary table of the chemical structures of nanomedicines.

Nanomedicines	Chemical Formula	Chemical Formula	Ref.
Chlorin e6	C_33_H_36_N_4_O_6_	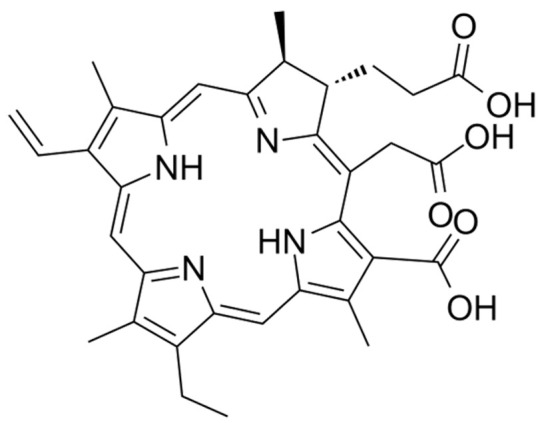	[68]
IR-780	C_36_H_44_ClIN_2_	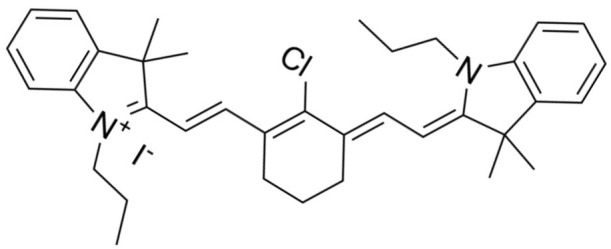	[32]
Sorafenib	C_21_H_16_ClF_3_N_4_O_3_	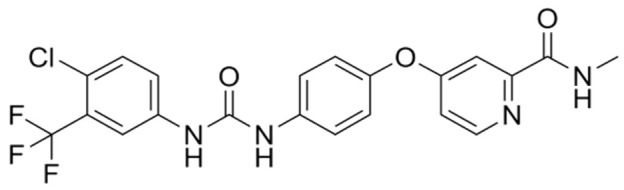	[68]
DOX	C_27_H_30_ClNO_11_	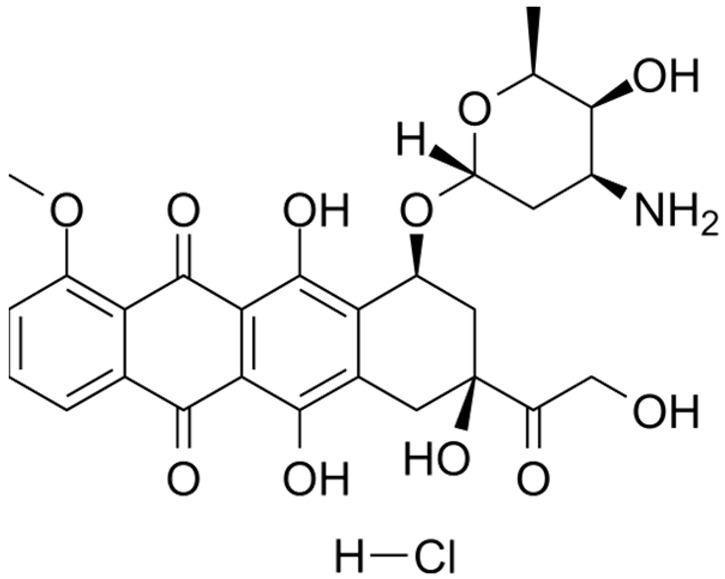	[71]
AIPH	C_12_H_24_C_l2_N_6_	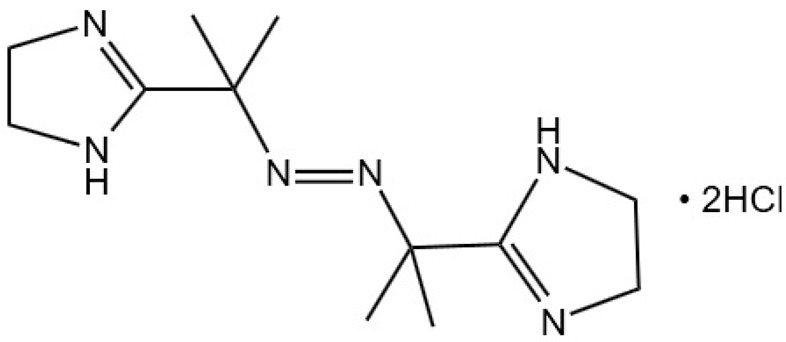	[41]
Verteporfin	C_82_H_84_N_8_O_16_	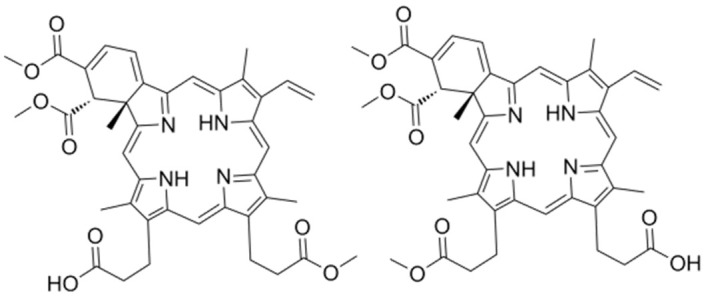	[100]
Tetrakis(4-carboxyphenyl) porphyrin	C_48_H_30_N_4_O_8_	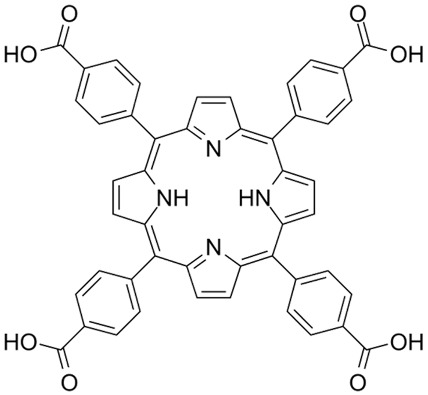	[44]
DON	C_6_H_9_N_3_O_3_	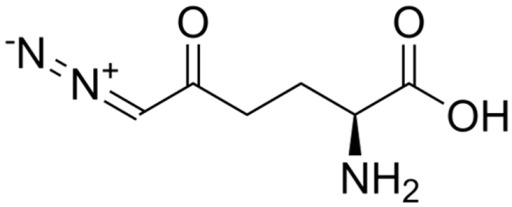	[35]
Cisplatin	C_l2_H_6_N_2_Pt	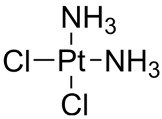	[114]

## Data Availability

No new data were created or analyzed in this study. Data sharing is not applicable to this article.
